# Modern Burn Surgery: Operative Decisions From Acute Excision to Long-Term Reconstruction

**DOI:** 10.7759/cureus.117564

**Published:** 2026-10-06

**Authors:** Daniel Gravino

**Affiliations:** 1 Plastic and Reconstructive Surgery, Mater Dei Hospital, Msida, MLT

**Keywords:** burns, burn surgery, dermal substitutes, evidence-based medicine, excision, reconstruction, scar surgery, skin grafting

## Abstract

Burn injury remains a major global surgical and public health problem, causing substantial mortality, disability, economic loss, and long-term functional impairment, with a disproportionate burden in low- and middle-income countries. This narrative evidence-based review critically examines contemporary burn surgery across the entire burn care pathway, from initial assessment and operative timing to excision, wound coverage, reconstruction, scar surgery, and emerging technologies, with emphasis on evidence quality and ongoing controversies. A comprehensive search of PubMed, EMBASE, and the Cochrane Library was conducted for English-language publications from 2000 to 2026 using search terms related to burn surgery, burn wound management, excision, skin grafting, dermal substitutes, reconstruction, and scar management. Priority was given to international guidelines, systematic reviews, meta-analyses, randomized controlled trials, cohort studies, and landmark surgical publications. Current evidence supports early specialist assessment, timely excision of clearly deep burns, and definitive wound closure with autologous skin grafting or selected adjunctive reconstructive techniques as the cornerstones of modern burn surgery. However, uncertainty persists regarding the optimal timing of excision, the comparative effectiveness of debridement techniques, and the indications for dermal substitutes and regenerative therapies. Increasing evidence also supports a continuum-based reconstructive approach that integrates scar prevention and functional restoration from the acute phase of burn care. Overall, burn surgery has evolved from simple wound closure to biologically informed, function-preserving reconstruction across the entire burn care pathway. Although significant advances have been achieved, further high-quality comparative research is required to define optimal surgical strategies and facilitate the safe integration of emerging regenerative technologies into routine clinical practice.

## Introduction and background

Burn injuries constitute a pervasive global surgical and public health crisis, presenting an immediate physiological threat compounded by a prolonged, often lifelong, reconstructive burden. While the scope of this review is surgical, effective prehospital first aid remains the critical initial intervention to limit burn wound conversion within the zone of stasis, thereby establishing the physiological foundation for subsequent surgical management [1-4].

Comprehensive global analyses estimate approximately nine million incident burn injuries and more than 110,000 burn-related deaths annually, with the greatest burden occurring in low- and middle-income countries where limited access to specialized burn services contributes to poorer outcomes and greater disability [5-8].

Surgical decision-making fundamentally influences survival, infection risk, metabolic response, scar quality, functional recovery, and long-term quality of life [9-11]. Contemporary burn surgery therefore emphasizes accurate burn depth assessment, timely excision of nonviable tissue, and early definitive wound coverage using reconstructive techniques tailored to burn severity, anatomical location, patient physiology, and available institutional resources [9,12].

Autologous split-thickness skin grafting remains the gold standard for definitive wound closure; however, advances in burn surgery have expanded the reconstructive armamentarium to include hydrosurgical debridement, enzymatic debridement, dermal regeneration templates, autologous skin cell suspension systems, cultured epithelial autografts, negative-pressure wound therapy, and microsurgical free tissue transfer [9,10]. Despite these developments, uncertainty persists regarding the optimal timing of excision, patient selection for emerging technologies, long-term cost-effectiveness, and the translation of promising regenerative therapies into routine clinical practice [10,13-15].

This narrative review critically evaluates contemporary evidence across the surgical management of burn injuries, synthesizing current literature relevant to both specialized burn centers and resource-limited healthcare systems where infrastructure, workforce capacity, and resource availability influence reconstructive decision-making. A comprehensive literature search was undertaken using PubMed, Embase, and the Cochrane Library to identify English-language studies published between January 2000 and March 2026. Priority was given to clinical practice guidelines, systematic reviews, meta-analyses, landmark randomized controlled trials, and high-quality observational studies, while isolated case reports and small retrospective case series were excluded. In addition to reviewing current surgical strategies, this article examines disparities in burn care delivery worldwide and discusses future directions in regenerative and reconstructive burn surgery [8,12,16-18].

## Review

Pathophysiology relevant to surgery

Burn injury is a dynamic biological process in which tissue viability continues to evolve after the initial thermal insult. The injury produces a central zone of irreversible coagulation, a surrounding zone of stasis that may either recover or progress to necrosis, and an outer zone of hyperemia that is generally viable but biologically active [1-4].

Zones of Jackson

The Jackson model remains the most useful clinical framework for understanding burn wound evolution. The zone of coagulation is irreversibly nonviable, the zone of stasis is ischemic but potentially salvageable, and the zone of hyperemia is inflamed yet usually recovers. Burn depth is therefore not fixed at presentation and may evolve over time, making serial clinical reassessment essential [14,19,20].

Wound Progression and Microcirculation

Progression of injury within the zone of stasis is driven by microvascular thrombosis, edema, leukocyte activation, inflammatory mediator release, and oxidative stress. Consequently, burns that initially appear superficial may deepen over several days. This biological process provides the rationale for optimizing tissue perfusion, oxygenation, temperature regulation, and infection prevention during the early phases of burn management [6,20,21].

Inflammatory Response

Major burns initiate profound local and systemic inflammatory responses characterized by increased vascular permeability, tissue edema, cytokine release, hypermetabolism, and immune dysregulation. These physiological alterations significantly influence wound healing, susceptibility to infection, and tolerance of subsequent surgical intervention [22,23].

Scar Biology

Hypertrophic scarring results from excessive fibroproliferation, persistent inflammation, prolonged myofibroblast activity, and abnormal mechanotransduction within the healing dermis. The duration for which a deep burn remains unhealed is strongly associated with an increased risk of hypertrophic scar formation, reinforcing the importance of timely definitive wound closure. Emerging evidence suggests that, particularly in pediatric populations, scar quality is also influenced by patient age, anatomical site, and other biological factors [16,24,25].

Healing Mechanisms

Superficial and selected partial-thickness burns heal through epithelial migration from preserved keratinocyte reservoirs within hair follicles and sweat glands. In contrast, deep dermal and full-thickness burns lack sufficient regenerative capacity and therefore require surgical excision followed by skin grafting or alternative reconstructive techniques [14,26-28].

Initial surgical assessment

Initial assessment establishes both immediate lifesaving priorities and the definitive surgical pathway. Appropriate prehospital first aid, particularly cooling the burn with 20 minutes of cool running water, limits burn wound progression within the zone of stasis and improves tissue preservation before definitive surgical evaluation. Surgeons must accurately determine burn depth, estimate total body surface area (TBSA), identify inhalation injury and associated trauma, and distinguish wounds likely to heal conservatively from those requiring operative intervention. Because burn wounds evolve over time, this assessment should be repeated during the first several days after injury [29-34].

Clinical Depth Assessment

Clinical assessment relies on burn color, capillary refill, blanching response, pain, sensation, and wound moisture. However, bedside evaluation remains imperfect during the first 24-72 hours because burn depth may continue to evolve as injury progresses within the zone of stasis [19,32].

TBSA Estimation

TBSA is commonly estimated using the Rule of Nines in adults [35] and the Lund and Browder chart in children [36]. These estimates guide fluid resuscitation, referral decisions, and operative planning but remain subject to interobserver variability, particularly in irregular burns and patients with atypical body habitus. Computer-assisted planimetry, three-dimensional imaging, and smartphone-based assessment tools demonstrate improved accuracy but have not yet achieved widespread clinical implementation [37-45].

Referral and Severity Stratification

Modern burn pathways emphasize early referral of patients with deep, extensive, circumferential, complex, or inhalation-associated burns to specialized burn centers, where multidisciplinary expertise improves reconstructive outcomes and reduces preventable morbidity [12].

Imaging in burn depth estimation

Objective imaging technologies complement clinical examination by improving diagnostic accuracy in burns of indeterminate depth. These modalities assess tissue perfusion, oxygenation, or structural integrity to better predict wound healing potential and guide operative decision-making. Nevertheless, they remain adjuncts to, rather than replacements for, expert clinical assessment [39].

Recent developments in AI and large language model-assisted diagnostic systems have demonstrated diagnostic performance approaching that of experienced burn surgeons, suggesting an expanding role for digital decision-support systems in future burn care.

Laser Doppler Imaging (LDI)

LDI is widely regarded as the reference standard for noninvasive burn depth assessment because it objectively measures microvascular perfusion. Numerous studies have demonstrated superior diagnostic accuracy compared with clinical examination alone, particularly when predicting healing potential and identifying wounds likely to require surgery [29,38,39].

Other Imaging Modalities

Additional technologies, including infrared thermography, laser speckle contrast imaging, ultrasound, optical coherence tomography, and multispectral or hyperspectral imaging, have demonstrated promising diagnostic performance by evaluating tissue perfusion, oxygenation, or structural characteristics. AI-based image analysis and convolutional neural networks have further improved diagnostic accuracy and, in selected datasets, have achieved performance comparable to or exceeding experienced clinicians. Despite these advances, widespread implementation remains limited by cost, availability, portability, and technical complexity [39].

Limitations

No single imaging modality currently provides universal accuracy while remaining rapid, inexpensive, and widely accessible. LDI is susceptible to motion artifact, dressing interference, wound contamination, and exudate, while AI-based systems require careful validation across diverse patient populations to minimize algorithmic bias and ensure equitable diagnostic performance. Consequently, these technologies should be regarded as clinical decision-support tools that augment, rather than replace, comprehensive clinical assessment [39].

Timing of surgery

The timing of burn excision requires balancing the benefits of early source control against the patient’s physiological reserve. Early excision reduces the inflammatory burden, decreases microbial colonization, and facilitates definitive wound closure; however, surgery should only proceed once adequate hemodynamic stability has been achieved. Optimal timing therefore depends upon burn depth, injury severity, associated injuries, patient physiology, institutional resources, and the evolution of the zone of stasis [14,32,40].

Immediate and Ultra-Early Surgery

Immediate excision following physiological stabilization aims to rapidly eliminate devitalized tissue, reduce bacterial burden, limit systemic inflammation, and establish a viable wound bed suitable for definitive reconstruction [14,31,41].

Early vs. Delayed Excision

Early excision, generally performed within the first 24-48 hours after injury, remains the accepted standard for deep dermal and full-thickness burns. Removal of necrotic tissue decreases infectious complications, shortens hospital stay, improves graft take, and facilitates earlier rehabilitation. Delayed excision may nevertheless be appropriate in patients with physiological instability or when burn depth remains uncertain because of ongoing evolution within the zone of stasis. Contemporary evidence also suggests that, particularly in pediatric patients, long-term scar quality depends not only on surgical timing but also on factors including patient age, anatomical location, and burn severity [14,31,32,40,41].

Practical Recommendation

Current evidence supports individualized operative timing. Definitive deep-thickness burns should undergo early excision once the patient has achieved adequate physiological stabilization, whereas indeterminate burns require careful serial reassessment to avoid unnecessary excision of viable tissue or delayed treatment of evolving necrosis [31,32,40].

Burn wound excision

Burn wound excision converts an unstable, contaminated wound into a viable reconstructive defect by removing devitalized tissue, reducing bacterial burden, attenuating the systemic inflammatory response, and preparing an optimal wound bed for definitive closure. Depending on burn depth, anatomical location, and preservation of viable dermis, excision may be performed using tangential or fascial techniques. Early excision remains the cornerstone of modern burn surgery because it improves graft take, reduces infection, shortens hospital stay, and decreases mortality in appropriately selected patients [14,41,42]. The principal surgical excision techniques used in contemporary burn surgery, together with their indications, advantages, and limitations, are summarized in Table 1.

**Table 1:** Comparison of excision techniques. Sources: [40-44]

Technique	Selectivity	Advantages	Limitations	Typical role
Tangential excision	Moderate-high	Preserves viable dermis; standard approach	Blood loss; technically demanding	Mainstay for deep partial/full-thickness burns
Fascial excision	Low	Rapid; effective source control	Deforming; poorer cosmesis	Massive or unstable burns
Hydrosurgery	High	Selective, tissue-sparing; precise debridement	Cost; availability; learning curve	Mixed-depth, smaller wounds
Bromelain enzymatic debridement	Very high	Dermal preservation; may reduce the need for excision	Pain; cost; logistics	Deep partial/full-thickness burns

Skin grafting

Skin grafting remains the cornerstone of definitive coverage following excision, serving as the primary modality for closing deep burn wounds [3,14,42,46]. The selection of graft type is a nuanced decision, fundamentally dictated by donor-site availability, wound location, and requisite functional or aesthetic outcomes [42]. Split-thickness skin grafts are the workhorse for most deep burns, offering reliable take and the ability to cover large surface areas, though they are associated with increased rates of contracture and donor-site morbidity. Conversely, full-thickness skin grafts are favored for smaller, aesthetically sensitive, or functional defects; by transferring both epidermis and dermis, they provide superior elasticity and reduced contraction, although their utility is often constrained by limited donor-site availability [42]. The characteristics, indications, advantages, and limitations of the commonly used skin grafting techniques are summarized in Table 2.

**Table 2:** Skin graft options. Source: [42]

Graft option	Main indication	Advantages	Limitations
Split-thickness skin graft	Most deep burns after excision	Reliable take, extensive coverage	Contraction, donor morbidity
Full-thickness skin graft	Small functional/aesthetic defects	Better elasticity, less contraction	Limited donor availability
Meshed graft	Large surface area coverage	Expands donor skin	Poorer cosmesis
Sheet graft	Visible/functional areas	Best cosmesis	Requires more donor skin
Cultured epithelial autograft	Massive burns, limited donor skin	Donor-sparing	Time-consuming, fragile, resource-intensive
Dermal substitutes (e.g., biodegradable temporizing matrix, Integra)	Extensive burns, limited donor availability	Improved pliability, reduced contraction	Two-stage process, infection risk

Dermal substitutes

Dermal substitutes have become integral to modern burn reconstruction, effectively addressing the inherent limitations of split-thickness grafting by fostering the generation of a neodermis, which facilitates reduced contraction and improved long-term scar pliability [42]. The field has increasingly shifted toward synthetic, bioresorbable polymers, which may offer reduced infection risks compared to earlier-generation bovine collagen-based matrices. While clinical indications remain heterogeneous, these materials are particularly valuable in extensive injuries where donor-site availability is constrained, serving as a critical component in complex, staged wound management [42]. The most widely used dermal substitutes in modern burn reconstruction and their principal clinical applications are summarized in Table 3.

**Table 3:** Dermal substitutes. Source: [42]

Product/category	Main indication	Advantages	Limitations
Integra	Deep burns requiring staged dermal replacement	Established; improves pliability	Two-stage process; infection risk
NovoSorb biodegradable temporizing matrix	Complex burns and scar reconstruction	Synthetic; versatile	Emerging evidence
Acellular dermal matrices	Acute burns and reconstruction	Dermal support	Heterogeneous products
Collagen-elastin matrices	Deep dermal/full-thickness wounds	May improve elasticity	Delayed re-epithelialization reported

Flap reconstruction

Flap reconstruction is increasingly recognized as a strategic, integral component of modern burn care rather than a tertiary option [47]. While primary closure or skin grafting remains the standard for many wounds, flap coverage, ranging from local tissue rearrangements to microsurgical free flaps, is essential when addressing exposed critical structures such as tendons, nerves, or bone, or when donor-site limitations necessitate more durable options [42]. Recent technological advancements, including robot-assisted microsurgery, have significantly enhanced precision and addressed ergonomic challenges in complex reconstructive efforts, as evidenced by studies such as Struebing et al. The application of the reconstructive ladder allows for tailored solutions that prioritize long-term functional and aesthetic outcomes [48]. The major flap reconstructive options, including their indications, advantages, and limitations, are summarized in Table 4.

**Table 4:** Flap reconstruction options. Sources: [47,48]

Flap type	Indication	Advantages	Limitations
Local flap	Small defects with adjacent healthy tissue	Simple; tissue match	Limited by surrounding scarring
Regional flap	Moderate defects needing durable coverage	Reliable vascularity	Donor morbidity
Free flap	Large or critical defects	Maximum versatility	Microsurgical complexity; total free flap loss ≈9.9%, partial loss ≈4.8%

Evidence supporting free flap reconstruction in burns highlights high overall success rates, suggesting that technical complexity should not preclude its use in complex burn populations [45-47]. This proactive reconstructive mindset is further bolstered by the integration of robotics into microsurgical practice, with systems such as the Symani Surgical System demonstrating efficacy in complex reconstructive cases [49].

Escharotomy and fasciotomy

Circumferential full-thickness burns form a constrictive eschar that can critically impede venous return and arterial perfusion, while thoracic involvement may restrict chest wall excursion, necessitating urgent escharotomy to restore perfusion and ventilation. When injury involves deeper tissues, edema can lead to compartment syndrome, where elevated interstitial pressure within the muscle fascia jeopardizes tissue viability; in such instances, fasciotomy is mandatory to prevent irreversible muscle necrosis and permanent functional impairment. Clinical indications for these decompression procedures include loss of distal pulses, progressive paresthesia, elevated compartment pressures, and severe restriction of ventilatory mechanics [1,2,50].

Hand burn surgery

Hand burns are disproportionately disabling, frequently accounting for 80-90% of severe burn injuries, as even minor scarring can significantly compromise grip, pinch, and dexterity [51]. Given the profound impact of these injuries on daily function, management requires a rigorous approach beginning with early edema control and splinting to minimize subsequent contracture. A proactive reconstructive mindset is critical; clinicians should utilize the reconstructive ladder to inform early operative decision-making, opting for flap coverage when tendons or joints are exposed to ensure superior functional outcomes and durability compared with traditional grafting [42,47]. Surgical intervention for such defects should be prioritized within the first 72 hours, as early excision and coverage are associated with reduced infection rates and lower mortality in severe injuries [52-55]. In pediatric populations, however, nuance is warranted. In a small cohort of children with burns involving 1-14.9% total body surface area, Steinvall et al. found that time from injury to skin grafting was not independently associated with scar quality at 12 months, whereas younger age, anatomical site, and longer healing time after grafting were associated with poorer scar outcomes [56].

Facial burn reconstruction

Facial burns require reconstruction to restore both function and appearance, respecting facial subunits and dynamic movement across the eyelids, nose, lips, ears, and scalp. The reconstructive strategy prioritizes preservation of these critical aesthetic units, often necessitating the use of sheet grafts or regional pedicled flaps to prevent tethering contractures that could otherwise impair essential functions such as the blink reflex and oral competence [3,4].

Burn scar reconstruction

The management of burn scars represents a critical, longitudinal phase of reconstruction that extends beyond reactive intervention to optimize long-term functional and aesthetic outcomes. Addressing the persistent sequelae of deep burns, specifically hypertrophic scarring and debilitating contractures, requires a tailored, multidisciplinary approach. In pediatric populations, management necessitates specific attention to ongoing somatic growth, which may influence the timing of intervention relative to scar maturation and its predictability for long-term outcomes. For both pediatric and adult patients, the approach harmonizes traditional surgical techniques, such as Z-plasty, contracture release, and flap reconstruction, with regenerative modalities including fat grafting and laser therapy [3,47]. This phase serves as an essential bridge between acute wound closure and comprehensive rehabilitation, where the careful selection of surgical interventions is dictated by anatomic site, scar maturity, and the overarching goal of restoring patient quality of life [42]. In this context, standardized assessment includes the Brisbane Burn Scar Impact Profile (BBSIP), a patient-reported instrument developed to assess burn-scar-specific health-related quality of life, with versions for adults, children, and caregivers [57].

Modern technologies

AI offers transformative potential for burn depth assessment, triage, and outcome prediction, promising to significantly augment the decision-making capacity of clinicians. Recent benchmarks underscore this dual potential; for instance, emerging assessments of large language model performance vs. expert clinician reasoning, alongside high-accuracy convolutional neural network models for image-based wound depth estimation, suggest that algorithmic diagnostic precision may soon approach or exceed human performance in controlled settings. Despite this progress, clinical implementation remains constrained by a reliance on small, heterogeneous datasets and documented performance biases across diverse skin tones [54,55]. Achieving the full utility of these technologies requires a critical transition toward large-scale, multi-institutional validation and standardized data sharing to ensure equitable and robust clinical applicability [52].

In parallel, advancements in laser therapy and mechanical interventions necessitate a departure from the traditional, rigid dichotomy between “acute” and “reconstructive” surgical phases. Embracing a continuum of care, where reconstructive principles are proactively integrated from the time of injury, including the emerging role of robot-assisted microsurgery for its enhanced precision and ergonomic advantages, particularly in medically fragile patients, is essential to optimizing long-term functional and aesthetic outcomes [47,53]. Furthermore, microsurgical techniques, once reserved as a tertiary resort, are increasingly validated as viable, strategic tools for complex reconstruction when conventional autografting options are exhausted [49]. This evolution in the treatment paradigm reinforces the integral role of the plastic surgeon in navigating complex clinical trajectories to maximize both survival and long-term functional recovery [48,58-64]. As these therapeutic modalities continue to advance, the focus of research must shift toward defining standardized protocols that harmonize regenerative medicine with established reconstructive techniques [3,42].

Current guidelines

Standardized, biologically informed care pathways are essential for optimizing clinical outcomes across diverse settings. Key recommendations from major international burn care guidelines are summarized in Table 5 and highlight the importance of standardized, biologically informed care pathways to optimize clinical outcomes across diverse healthcare settings [3,42]. These efforts are particularly critical in the management of burn injuries, where the rapid evolution of regenerative strategies, such as the utilization of adipose-derived stem cells, demands robust, evidence-based frameworks to validate clinical efficacy.

**Table 5:** International guideline recommendations. Sources: [63-65]

Organization	Main recommendation	Practical implication
American Burn Association	Early specialist assessment, structured resuscitation	Supports early referral and protocolized care
British Burn Association	Standardized burn-center pathways	Reinforces centralized specialist management
International Society for Burn Injuries	Global burn care standards across resource settings	Applicable in high- and low-resource systems
Joint Trauma System	Definitive care via excision/grafting where feasible	Relevant for deployed/military and prolonged-transfer settings

Future directions

Achieving the next stage of advancement in burn care requires a strategic synthesis of technological innovation and clinical rigor. Central to this evolution is a shift toward a personalized reconstructive paradigm, tailored to specific wound biology and patient risk profiles, which must be underpinned by standardized outcome reporting, such as the BBSIP [55], now increasingly recognized as a key tool for longitudinal quality-of-life tracking, and by robust, prospective comparative trials to definitively establish evidence-based practice [59]. Establishing a common framework for validation across centers will be essential to allow for the effective, large-scale comparison of these emerging therapeutic interventions [60]. Furthermore, research must prioritize high-level evidence, such as randomized controlled trials, to move beyond current limitations in assessing both the clinical and economic impacts of new regenerative strategies [59]. Concurrent with these clinical initiatives, the development of fully functional, tissue-engineered composite skin remains the ultimate goal for restoring complex structural appendages and biological integrity [61,62]. The rapid pace of development between 2024 and 2026 necessitates that research frameworks remain highly adaptive to these fast-evolving therapeutic innovations.

Ongoing investigations into biomarker profiling and stem cell applications are expected to further refine inflammation control and facilitate more predictable graft integration, while simultaneously addressing the long-standing challenge of creating a multilayered, off-the-shelf substitute that incorporates essential elements such as vascularity, innervation, and sweat glands [58]. Additionally, the implementation of nanostructured scaffolds and smart stimuli-responsive materials promises to enhance the precision of antimicrobial delivery and the cellular microenvironment [63]. These innovations are further complemented by cell-spraying technologies and bioprinting, which, despite requiring specialized clinical infrastructure, provide unprecedented control over cellular delivery to optimize re-epithelialization in deep partial-thickness injuries [64-68].

## Conclusions

Burn surgery has matured into an increasingly specialized and integrative discipline, encompassing acute source control, definitive wound closure, function-preserving reconstruction, and comprehensive long-term scar management. While current evidence unequivocally supports early specialist assessment and prompt closure of clearly deep burns to optimize initial outcomes, significant technical and timing questions persist. The most pressing unmet need remains the generation of robust, prospective comparative outcome data that accurately reflect diverse real-world patient populations, a critical step toward establishing definitive evidence-based practice. Furthermore, fostering a multidisciplinary approach that synergizes preclinical innovations with clinical expertise will be pivotal in reducing patient morbidity, optimizing hospital resource utilization, and ultimately improving long-term quality of life. As the discipline moves forward, the harmonization of emerging technologies, ranging from advanced scaffold design to AI-driven diagnostic tools, will be essential to transition from reactive treatment models toward a proactive, patient-centered paradigm. Implementation strategies must prioritize hybrid models in which AI serves as an adjunct to the expert clinician rather than a replacement; integrating high-speed segmentation with the contextual judgement of the plastic surgeon is critical to ensuring patient-centered outcomes that prioritize functional preservation and aesthetic refinement. Achieving this objective necessitates sustained commitment to both technological refinement and clinical training, ensuring that the next generation of practitioners can effectively leverage multidisciplinary advancements in wound repair and functional reconstruction.

## References

[1] Asuku M, Shupp JW (2023). Burn wound conversion: clinical implications for the treatment of severe burns. J Wound Care.

[2] Hettiaratchy S, Dziewulski P (2004). ABC of burns: pathophysiology and types of burns. BMJ.

[3] Smolle C, Cambiaso-Daniel J, Forbes AA (2017). Recent trends in burn epidemiology worldwide: a systematic review. Burns.

[4] Hu Y, Zhi L, Wang Y (2025). Global, regional, and national burden of burns from 1990 to 2021: a systematic analysis of prevalence, incidence, mortality, and disability-adjusted life years with projections to 2036. J Craniofac Surg.

[5] Lee N, Bae Y, Jang S, Lee DW, Lee SW (2025). Global, regional, and national burden of burn injury by total body surface area (TBSA) involvement from 1990 to 2021, with projections of prevalence to 2050. Healthcare (Basel).

[6] Lu S, Yakupu A, Zhang J, Dong W, Song F, Dong J (2022). The epidemiology and trends in the burden of burns throughout the world [PREPRINT]. Res Sq.

[7] Gerstl J, Kilgallon J, Nawabi N (2021). The global macroeconomic burden of burn injuries. Plast Reconstr Surg Glob Open.

[8] Żwierełło W, Piorun K, Skórka-Majewicz M, Maruszewska A, Antoniewski J, Gutowska I (2023). Burns: classification, pathophysiology, and treatment: a review. Int J Mol Sci.

[9] Neriamparambil AJ, Sawhney R, Wong WL (2025). Evidence-based management of burns: a narrative review of evolving practices. Eur Burn J.

[10] Rowan MP, Cancio LC, Elster EA (2015). Burn wound healing and treatment: review and advancements. Crit Care.

[11] Egelko A, Stewart B, Curtis E, Nsaful K, Rae L, Yadav M (2026). Explosive Weapons Trauma Care Collective (EXTRACCT) Blast Injury Clinical Practice Guideline: burn management in low-resource settings. World J Surg.

[12] Amini-Nik S, Yousuf Y, Jeschke MG (2018). Scar management in burn injuries using drug delivery and molecular signaling: Current treatments and future directions. Adv Drug Deliv Rev.

[13] Forbinake NA, Ohandza CS, Fai KN, Agbor VN, Asonglefac BK, Aroke D, Beyiha G (2020). Mortality analysis of burns in a developing country: a CAMEROONIAN experience. BMC Public Health.

[14] Najafali D, Rezaei S, Liu H (2025). 953 Global disparities in burns: analyzing outcomes from the WHO Global Burn Registry across resource settings. J Burn Care Res.

[15] Hardaway RM 3rd (1969). Clinical management of shock. Mil Med.

[16] Toussaint J, Singer AJ (2014). The evaluation and management of thermal injuries: 2014 update. Clin Exp Emerg Med.

[17] Lu M, Zhao J, Wang X, Zhang J, Shan F, Jiang D (2022). Research advances in prevention and treatment of burn wound deepening in early stage. Front Surg.

[18] Evers LH, Bhavsar D, Mailänder P (2010). The biology of burn injury. Exp Dermatol.

[19] Jeschke MG, Chinkes DL, Finnerty CC (2008). Pathophysiologic response to severe burn injury. Ann Surg.

[20] Finnerty CC, Jeschke MG, Branski LK, Barret JP, Dziewulski P, Herndon DN (2016). Hypertrophic scarring: the greatest unmet challenge after burn injury. Lancet.

[21] Taylor ZD, Singh RS, Bennett DB (2011). THz medical imaging: in vivo hydration sensing. IEEE Trans Terahertz Sci Technol.

[22] Gauglitz GG, Korting HC, Pavicic T, Ruzicka T, Jeschke MG (2011). Hypertrophic scarring and keloids: pathomechanisms and current and emerging treatment strategies. Mol Med.

[23] Bello YM, Phillips TJ (2000). Recent advances in wound healing. JAMA.

[24] Concannon E, Damkat-Thomas L, Coghlan P (2022). Role of skin substitutes in burn wound reconstruction. Wound Healing - Recent Advances and Future Opportunities.

[25] Kartal SP, Altunel CT, Bayramgürler D (2018). Introductory chapter: an introduction to burn injuries. Hot Topics in Burn Injuries.

[26] Burgess M, Valdera F, Varon D, Kankuri E, Nuutila K (2022). The immune and regenerative response to burn injury. Cells.

[27] Irilouzadian R, Khalaji A, Baghsheikhi H, Sarmadian R, Hoveidamanesh S, Ghadimi T, Farokh Forghani S (2023). The clinical outcomes of xenografts in the treatment of burn patients: a systematic review and meta-analysis. Eur J Med Res.

[28] Burmeister DM, Ponticorvo A, Yang B, Becerra SC, Choi B, Durkin AJ, Christy RJ (2015). Utility of spatial frequency domain imaging (SFDI) and laser speckle imaging (LSI) to non-invasively diagnose burn depth in a porcine model. Burns.

[29] Monstrey S, Hoeksema H, Verbelen J, Pirayesh A, Blondeel P (2008). Assessment of burn depth and burn wound healing potential. Burns.

[30] Han G, Ceilley R (2017). Chronic wound healing: a review of current management and treatments. Adv Ther.

[31] Nicks BA, Ayello EA, Woo K, Nitzki-George D, Sibbald RG (2010). Acute wound management: revisiting the approach to assessment, irrigation, and closure considerations. Int J Emerg Med.

[32] Murari A, Singh KN (2019). Lund and Browder chart-modified versus original: a comparative study. Acute Crit Care.

[33] Alharbi Z, Piatkowski A, Dembinski R, Reckort S, Grieb G, Kauczok J, Pallua N (2012). Treatment of burns in the first 24 hours: simple and practical guide by answering 10 questions in a step-by-step form. World J Emerg Surg.

[34] Tremblay C, Albrecht KA, Sonke CC, Azad S (2024). Approach to burn treatment in the rural emergency department. Can Fam Physician.

[35] Wallace AB (1951). The exposure treatment of burns. Lancet.

[36] Rumpf RW, Stewart WC, Martinez SK (2018). Comparison of the Lund and Browder table to computed tomography scan three-dimensional surface area measurement for a pediatric cohort. J Surg Res.

[37] Li S, Mohamedi AH, Senkowsky J, Nair A, Tang L (2020). Imaging in chronic wound diagnostics. Adv Wound Care (New Rochelle).

[38] Carrière ME, de Haas LE, Pijpe A, Meij-de Vries A, Gardien KL, van Zuijlen PP, Jaspers ME (2020). Validity of thermography for measuring burn wound healing potential. Wound Repair Regen.

[39] Álvarez GE, Contreras JE, Jiménez EM (2025). Advanced reconstructive strategies in modern burn care: an integrated, multidisciplinary review. Int J Med Sci Clin Res Stu.

[40] Matsumura H, Nozaki M, Watanabe K (2012). The estimation of tissue loss during tangential hydrosurgical debridement. Ann Plast Surg.

[41] Yeh CC, Lin YS, Hsu YC (2008). The Versajet™ Hydrosurgery System-managed burn wound with reduced scar contracture [Article in Chinese]. J Chinese Med Assoc.

[42] De Freitas LR, Udoma-Udofa OC, Reginato PH, Mitsui HC, Provinciatto H, Torres FC (2025). Bromelain-based enzymatic debridement versus standard of care in deep burn injuries: a systematic review and meta-analysis. J Burn Care Res.

[43] Toledo YR, Gaviño UZ (2026). State of the art in burn care: principles and advances in surgical management. Int Surg J.

[44] Sheckter CC (2022). Unwinding the false paradigm of acute versus reconstructive management of burn injuries. Plast Reconstr Surg.

[45] Arumugam KL, Chittoria RK, Amrutha JS (2025). Role of dermabrasion assisted tangential excision in burns. Int J Med Sci Clin Res Stud.

[46] Fan C, Azam F, Hinson C (2025). Free flap reconstruction in burns: a systematic review of current practices and evidence. Microsurgery.

[47] Matsen FA 3rd, Winquist RA, Krugmire RB Jr (1980). Diagnosis and management of compartmental syndromes. J Bone Joint Surg Am.

[48] Struebing F, Bigdeli A, Weigel J (2024). Robot-assisted microsurgery: lessons learned from 50 consecutive cases. Plast Reconstr Surg Glob Open.

[49] Ar AY, Yiğit Y (2023). Treatment and follow-up for earthquake victims in the intensive care unit. Cam Sakura Med J.

[50] Donaldson J, Haddad B, Khan WS (2014). The pathophysiology, diagnosis and current management of acute compartment syndrome. Open Orthop J.

[51] Watzinger N, Hecker A, Petschnig D (2024). Long-term functional outcomes after hand burns: a monocentric controlled cohort study. J Clin Med.

[52] Osuna JA, Chavez VG, Castaneda JA (2025). Surgical management of burn injuries: current concepts and advancements in reconstructive strategies. Int Surg J.

[53] Moiemen N, Mathers J, Jones L (2018). Pressure garment to prevent abnormal scarring after burn injury in adults and children: the PEGASUS feasibility RCT and mixed-methods study. Health Technol Assess.

[54] Boissin C, Laflamme L, Fransén J (2023). Development and evaluation of deep learning algorithms for assessment of acute burns and the need for surgery. Sci Rep.

[55] Bhattachan P, Ricciuti Z, Khalaf F, Jeschke MG (2026). The role of artificial intelligence in burn assessment, complication diagnosis, and outcome prediction: a narrative review. Burns Trauma.

[56] Steinvall I, Kennedy S, Karlsson M (2025). Evaluating scar outcomes in pediatric burn patients following skin grafting. Sci Rep.

[57] Tyack Z, Ziviani J, Kimble R, Plaza A, Jones A, Cuttle L, Simons M (2015). Measuring the impact of burn scarring on health-related quality of life: development and preliminary content validation of the Brisbane Burn Scar Impact Profile (BBSIP) for children and adults. Burns.

[58] Kao CC, Garner WL (2000). Acute burns. Plast Reconstr Surg.

[59] Zuo KJ, Medina A, Tredget EE (2017). Important developments in burn care. Plast Reconstr Surg.

[60] E Moura FS, Amin K, Ekwobi C (2021). Artificial intelligence in the management and treatment of burns: a systematic review. Burns Trauma.

[61] Stone Ii R, Natesan S, Kowalczewski CJ (2018). Advancements in regenerative strategies through the continuum of burn care. Front Pharmacol.

[62] Chua AW, Khoo YC, Tan BK, Tan KC, Foo CL, Chong SJ (2016). Skin tissue engineering advances in severe burns: review and therapeutic applications. Burns Trauma.

[63] Singer AJ, Boyce ST (2017). Burn wound healing and tissue engineering. J Burn Care Res.

[64] Mofazzal Jahromi MA, Sahandi Zangabad P, Moosavi Basri SM (2018). Nanomedicine and advanced technologies for burns: preventing infection and facilitating wound healing. Adv Drug Deliv Rev.

[65] Shpichka A, Butnaru D, Bezrukov EA (2019). Skin tissue regeneration for burn injury. Stem Cell Res Ther.

[66] Boyce ST, Lalley AL (2018). Tissue engineering of skin and regenerative medicine for wound care. Burns Trauma.

[67] Kumar R, Keshamma E, Kumari B (2022). Burn injury management, pathophysiology and its future prospectives. J Res Appl Sci Biotechnol.

[68] Guan H, Zhang DL (2025). Multidisciplinary integration in the diagnosis and treatment in burns and wound repair surgery: current status, challenges, and future [Article in Chinese]. Zhonghua Shao Shang Yu Chuang Mian Xiu Fu Za Zhi.

